# Perspective: A Comprehensive Evaluation of Data Quality in Nutrient Databases

**DOI:** 10.1016/j.advnut.2023.02.005

**Published:** 2023-02-25

**Authors:** Zhaoping Li, Shavawn Forester, Emily Jennings-Dobbs, David Heber

**Affiliations:** 1 Center for Human Nutrition, David Geffen School of Medicine at University of California Los Angeles, Los Angeles, CA, United States; 2 Nutrient Institute LLC, Reno, NV, United States

**Keywords:** food composition data, nutrient data, data quality, essential nutrients, phytonutrients, omics, anthropometrics, human nutrition, precision nutrition, personalized dietary recommendations

## Abstract

Nutrient databases are a critical component of nutrition science and the basis of exciting new research in precision nutrition (PN). To identify the most critical components needed for improvement of nutrient databases, food composition data were analyzed for quality, with completeness being the most important measure, and for FAIRness, how well the data conformed with the data science criteria of findable, accessible, interoperable, and reusable (FAIR). Databases were judged complete if they provided data for all 15 nutrition fact panel (NFP) nutrient measures and all 40 National Academies of Sciences, Engineering, and Medicine (NASEM) essential nutrient measures for each food listed. Using the gold standard the USDA standard reference (SR) Legacy database as surrogate, it was found that SR Legacy data were not complete for either NFP or NASEM nutrient measures. In addition, phytonutrient measures in the 4 USDA Special Interest Databases were incomplete. To evaluate data FAIRness, a set of 175 food and nutrient data sources were collected from worldwide. Many opportunities were identified for improving data FAIRness, including creating persistent URLs, prioritizing usable data storage formats, providing Globally Unique Identifiers for all foods and nutrients, and implementing citation standards. This review demonstrates that despite important contributions from the USDA and others, food and nutrient databases in their current forms do not yet provide truly comprehensive food composition data. We propose that to enhance the quality and usage of food and nutrient composition data for research scientists and those fashioning various PN tools, the field of nutrition science must step out of its historical comfort zone and improve the foundational nutrient databases used in research by incorporating data science principles, the most central being data quality and data FAIRness.

**Statement of significance**

This review demonstrates for the first time that despite important contributions from the USDA and others, food and nutrient databases in their current forms do not yet provide truly comprehensive food composition data. We propose that to enhance the quality, accessibility, and utilization of food and nutrient composition data, data science principles should be incorporated into nutrient databases, the most central being data quality and FAIR criteria, which are “findable, accessible, interoperable, and reusable.”

## Introduction

**Figure 1:**
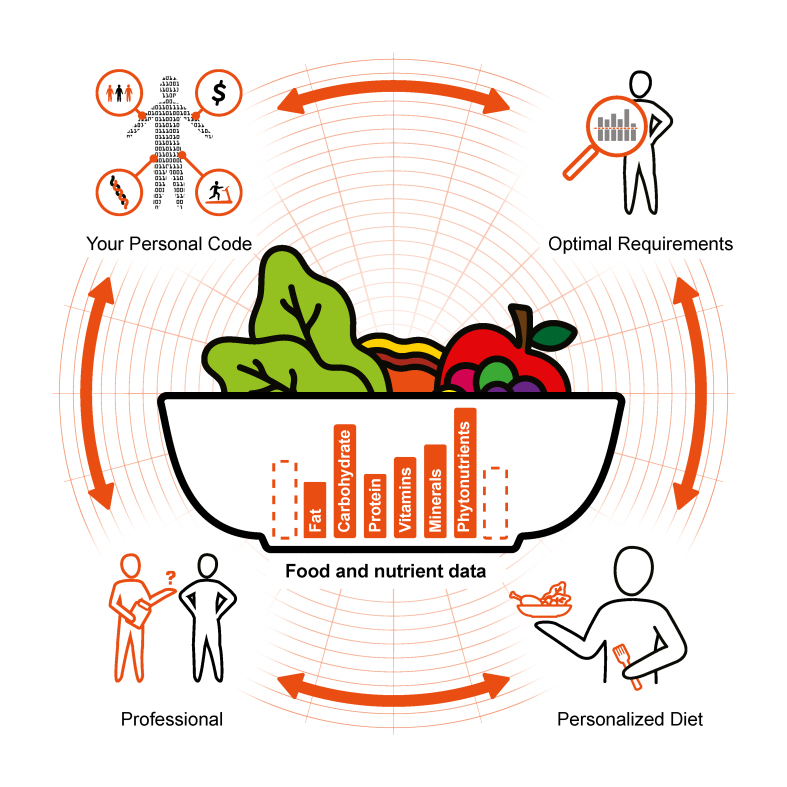
The precision nutrition (PN) network. PN is shown in this figure as a synergistic network with food composition data at its core. Bidirectional arrows connecting the icons represent the constant flow of both PN and food and nutrient data. Over time, these relationships remain tightly connected as data expand, understanding improves, and an individual changes. The “professional” icon represents experts in nutrition and medicine and AI-based algorithms that work directly with an individual. “Your personal code” represents all descriptive data, including but not limited to genomics, metabolomics, and human and microbiota phenomics, along with social and anthropometric data on eating behavior, physical activity, and economic and demographic data. “Optimal requirements” are specific nutrient requirements for an individual and are determined by one’s personal code. Personalized dietary recommendations, or a “personalized diet,” are the goal of PN, wherein the professional, the individual’s personal code, and optimal requirements are factored into a dietary plan best suited to provide the best tasting, achievable diet for optimal health.

Nutrition scientists are working toward more personalized nutrition recommendations based on an integration of insights from the new “omic” sciences, and a nutrient analysis of foods informed by nutrient databases are a key foundation essential to these efforts (Figure 1). Precision nutrition (PN) aims to assess an individual’s response to specific foods or dietary patterns and, thereby, determine the most effective diet interventions to prevent or treat specific diseases and conditions. Personalizing nutrition recommendations rather than a one-size-fits-all approach may increase the likelihood that individuals will comply with nutritional recommendations [1,2]. This individualized approach is accomplished by harmonizing food and nutrient data (also called “food composition data”) with modern omics technologies, such as genomics, transcriptomics, proteomics, metabolomics, metagenomics, and human and microbiota phenomics, along with information on eating behavior, physical activity, social, economic, and demographic data [[3], [4], [5]]. Food composition data are essential for executing PN and should aim to achieve comprehensiveness and quality comparable with omics data [6].

The NIH has initiated the largest PN study ever conducted, which will recruit 10,000 participants from the All of Us network. The goal is to use genetic and phenotypic medicine in addition to a wide range of omic measures and other individual factors such as education, employment status, and ZIP code to inform more precise nutrition recommendations. This endeavor has the potential to provide a rich data source that will inform dietary recommendations for the next decade and beyond [7]. The NIH Nutrition for Precision Nutrition Working Group has addressed some of the major challenges in dietary intake data collection, such as problems with self-reported dietary intake tools such as 24-h recalls [8]. Unfortunately, to date, this and other similarly ambitious science has not focused on the quality of food composition data regarding nutrient and micronutrient composition.

Historically, nutrition science has built knowledge on food characteristics that could then be used to develop dietary recommendations. Food and nutrient databases have been essential to those efforts, and nutrition science made great strides with an initially limited set of tools. These databases provide the chemical composition of food and beverage items as obtained from chemical analyses, estimations from published literature, or unpublished laboratory reports [9]. Comprehensive analysis and documentation of the micronutrient and macronutrient compounds in the food supply are foundational in determining dietary intake and health-promoting or harming properties of food components.

The USDA particularly has performed seminal work in developing food composition databases. The USDA food and nutrient databases are considered a gold standard and provide the data used globally for food and nutrition research. Housed within the USDA FoodData Central (FDC), these core data sources include 5 distinct data types, each with a particular purpose and unique attributes: the National Nutrient Database for SR Legacy Release (SR Legacy), the USDA Global Branded Food Products Database (Branded Foods), the Food and Nutrient Database for Dietary Studies (FNDDS), Foundation Foods, and Experimental Foods. Of those 5, SR Legacy is the most comprehensive food composition data source maintained by the USDA. It contains a total of 7793 foods and 149 food measures [10].

With the growth of scientific knowledge over the last century and a quarter, it has become clear that foods and nutrients are themselves complex and have complex interactions with the human physiology. However, food composition databases in their current forms do not provide truly comprehensive food and nutrient data. Although the USDA SR Legacy identifies 149 nutrients and other components, not all of these are indicated for each food. Moreover, plants produce >200,000 metabolites [11] and 50,000 secondary metabolites [12]. Many of these unmeasured or less-measured compounds in foods include micronutrients such as polyphenols, terpenes (which give many foods flavor and aroma), and various pigments [13]. These essential and nonessential food components modify a number of cellular processes associated directly with health including carcinogen metabolism, hormonal balance, cell signaling, apoptosis, and more [14]. They are also indirectly associated with health through their effect on gut microbiota and subsequent bacterial metabolites.

Nutrition scientists have historically focused on investigating biochemistry, food composition, and behavior and, to a lesser degree, nutrient database management. For example, nutrition science does not currently provide standards for what constitutes data source quality or how data should be managed. To address this problem, a greater interdisciplinary collaboration with data scientists and the implementation of data science principles in the management of food and nutrient data are needed to ensure that high-quality data are available for advancing PN. Two key data science principles of data quality and FAIRness will be critical in advancing the goals of PN [15,16].

Completeness and accuracy are primary indicators of data quality. Regarding food and nutrient data, the relevant metric for completeness is the number of available nutrient measures. An initial assessment of accuracy can be obtained from the age and update frequency of each data measure. These attributes are relevant because methods change and evolve over time such that older data may not reflect the present day nutrient content of foods [6].

The major recommendation emerging from this prospective review is the specific application of FAIR principles to food and nutrient data. Data FAIRness refers to how well data aligns with the FAIR principles of findable (F), accessible (A), interoperable (I), and reusable (R). The FAIR principles offer considerable value because they move beyond measuring data quality to evaluating how data itself are treated. The FAIR principles are particularly applicable to PN because they, similar to PN, involve bridging human-driven and machine-driven activities (for exmaple, big data). The FAIR approach combines and extends previous work by the Concept Web Alliance partners (focused on machine-actionability and harmonization of data structures and semantics) [17] and by the scientific and scholarly organizations that developed the Joint Declaration of Data Citation Principles (focused on making primary scholarly data citable, discoverable, and available for reuse, so that such data can support more rigorous scholarship) [18].

### Findable

Findability refers to how easily a data source can be located. Data sources are most often found by entering available metadata such as the data source name into a search engine. Metadata is all information about data—any information that characterizes a data source. A metadata record gives the basic “who, what, where, and when” of the relevant data. For instance, the publisher, release date, and temporal coverage of a data source are all different types of metadata. Thus, from a data science perspective, citation is the sharing of metadata required to find a given data source such as a website, publication, software program, database, or specific record within a database.

The most findable data sources, such as data published in a scientific journal or the USDA data, have unique persistent identifiers such as DOIs or static URLs. Data findability affects data usability: if a data source cannot be located despite a thorough web search, the data it contains cannot be used. Similarly, if a data source can only be found after intensive searching, it is less likely to be used in future research, potentially skewing future results.

### Accessible

Accessibility refers to how easily data can be acquired from a data source. The major factors that affect accessibility are money, authorization, and format. Money and authorization are binary: one can either pay or not pay; one either has authorization to access data or does not have authorization. Formatting is more difficult. One may be able to acquire data, but the format in which the data are exported may either facilitate or severely limit its usage.

The most accessible formats can be seamlessly imported into a basic analysis software that is widely available and requires minimal technical expertise to use, such as Microsoft Excel or comma-separated values (CSV). Formats such as SAS or Microsoft Access are less accessible because the corresponding software is more specialized and less widely available. To view data in those formats, users need to have a license to the specific software and have the technical expertise to operate the analytics software. The least accessible formats are images or PDFs that must be manually recorded into formats compatible with an analysis software or that require additional software to convert them into a compatible format for data extraction.

### Interoperable

Interoperability refers to the ability to harmonize multiple sources into a usable network. Interoperability is most often established by unique identifiers that can be used to directly link data sources or by the implementation of ontologies such as “LanguaL” [19] and “FoodEx2” [20], which can be used to identify different names of the same food or specimen.

However, the existence of a unique identifier alone is insufficient to make data sources interoperable. One also needs to know how to retrieve identifiers and link the sources or how to construct a useful ontology, even if that means simply knowing which niche software program to use to link identifiers. Building that know-how requires documentation and instructions for how to link and integrate relevant data sources.

### Reusable

Reusability depends heavily on the use of metadata. It is the primary responsibility of the data supplier to provide the metadata and the researcher to publish a comprehensive citation. The International Association for Social Science Information Services and Technology (IASSIST) has published citation guidelines [21], a set of established and highly regarded publication standards for managing data. The IASSIST citation guidelines go beyond citation style to establish the minimum elements of a citation that are necessary to identify and retrieve data used in research. The guidelines define 5 principal elements, the 2 most important of which are the full title of the data set and the electronic location. If the title and locator are not specific to the exact instance of the data used, citations must be appended with a retrieval date.

In this review, the current food and nutrient data landscape was analyzed to propose methods and data science principles that can ultimately ensure robust, reproducible nutrient data are available to advance PN. Data completeness and accuracy from the existing SR Legacy database were used as a representative example and additional analyses were performed using FAIR principles. Specific recommendations for improving the overall quality and FAIRness of food and nutrient composition data were proposed. Improvements in nutrient databases will be essential for research scientists and those fashioning various PN tools, including algorithmically based approaches to individualized dietary recommendations.

## Methods

### Identification and download of the USDA food and nutrient data

SR Legacy was downloaded in JavaScript Object Notation format from FDC on 1 December, 2021 and was read into the R software (version 1.8.0). Completeness was defined as the presence of reported values for all nutrition fact panel (NFP) measures mandated by the FDA standard labeling laws plus the presence of reported values for all essential nutrients as defined by the National Academies of Sciences, Engineering, and Medicine (NASEM), formerly the of Institute of Medicine, Dietary Reference Intake reports. MyPlate food groups were taken directly from the USDA dietary guideline reports [[22], [23], [24]].

In addition, phytonutrient data were collected from the most current versions of the 4 USDA Special Interest Databases relating to flavonoids, proanthocyanidins, and isoflavones from the USDA Agricultural Research Service website on 4 October, 2022 [[25], [26], [27], [28]]. These databases were used to acquire information on 38 phytonutrient measures: 4 flavonols, 2 flavones, 3 flavanones, 11 flavan-3-ols, 6 anthocyanidins, 5 proanthocyanidins, and 7 phytoestrogens. Phytonutrient classes were provided in the PDF documentation of each database. Duplicate nutrient measures were removed before calculation.

### Search strategy and publication selection process

**Figure 2:**
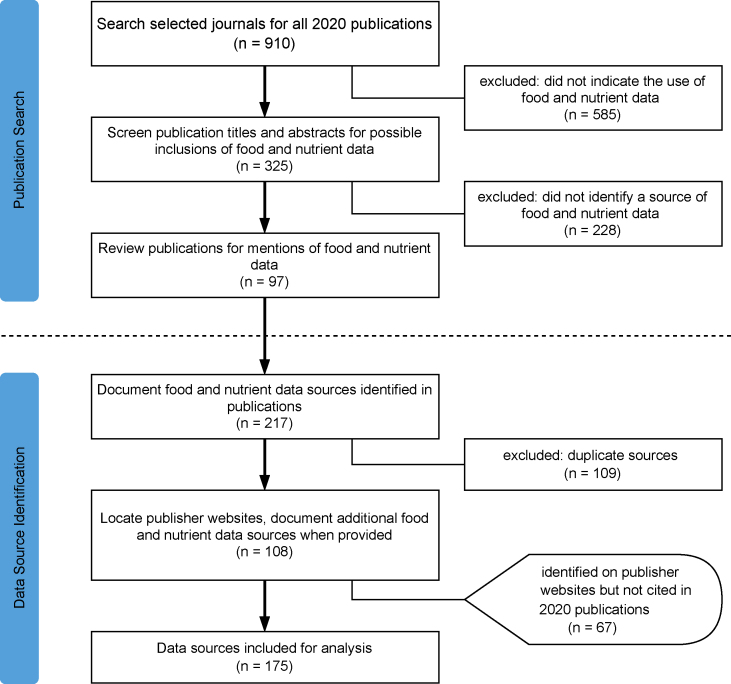
The publication search strategy and food and nutrient data selection process. Current food and nutrient data sources were identified, searched, and screened using a 2-part process—a publication search, followed by identification of food and nutrient data sources for subsequent analysis. Publications from the year 2020 were gathered from the top 5 nutrition and dietetics journals and were reviewed for mentions of food and nutrient data according to the steps shown. Food and nutrient data sources documented in the publications and organically identified from related sources were collected and documented according to the steps shown.

Current food and nutrient data sources were identified, searched, and screened according to the flowchart shown in Figure 2. The SCImago Journal Ranking (SJR) framework [29] was selected as an inclusion criteria tool. Based on this ranking method, the top 5 journals in the category nutrition and dietetics as ranked by the SJR in May 2021 were as follows: *1*) *The American Journal of Clinical Nutrition*, *2*) *Advances in Nutrition*, *3*) *Annual Review of Nutrition*, *4*) *International Journal of Behavioral Nutrition and Physical Activity*, and *5*) *Nutrition Reviews*. For each of the 5 journals, a publication search was performed using the search engine on the journal website and filtering for publications in the year 2020. The year 2020 was specified to identify the most current use of food and nutrient data at the time of collection. A total of 910 publications from the year 2020 were identified from the 5 journals.

A manual review of the 910 publications was initiated on 28 May, 2021. Publications were identified as most likely using food and nutrient data by screening the following keywords in either the title or abstract: “nutrients,” “nutrient density,” “nutrient profiling,” “nutrient composition,” “diet quality,” “nutrient values,” “macronutrients,” “micronutrients,” “vitamins,” “minerals,” “diet patterns,” “diet,” “meals,” “snacks,” “drinks,” “food,” “nutritional aspects,” “nutrient content,” “nutrition content,” “nutrient timing,” “nutrition requirements,” “dietary requirements,” “dietary behaviors,” “food behaviors,” “database,” “nutrient database,” “food database,” “weight loss,” “food data,” “nutrient data,” “food composition,” and “composition data.” Of the 910 publications, 325 were identified for a full-text review.

The 325 publications identified during screening were reviewed in detail by downloading and reading each publication in its entirety (including footnotes, tables, and figures). If supplemental data were identified as containing ≥1 source of further information about food and nutrient data, the supplemental data were also downloaded and reviewed. In total, 97 publications provided ≥1 source of food and nutrient data. Some publications provided >1 source.

### Identification of food and nutrient data sources

Trained data analysts collected data sources and corresponding metadata directly from the aforementioned 97 publications identified. Additional food and nutrient data sources were identified and collected because they were discovered during the data source analysis. Data characteristics were collected directly from publisher websites. Relevant data were recorded such as the title, data type, country of origin, format, accessibility, and inclusion of USDA data.

## Results

### Completeness analysis of the USDA SR Legacy food and nutrient data

**Figure 3:**
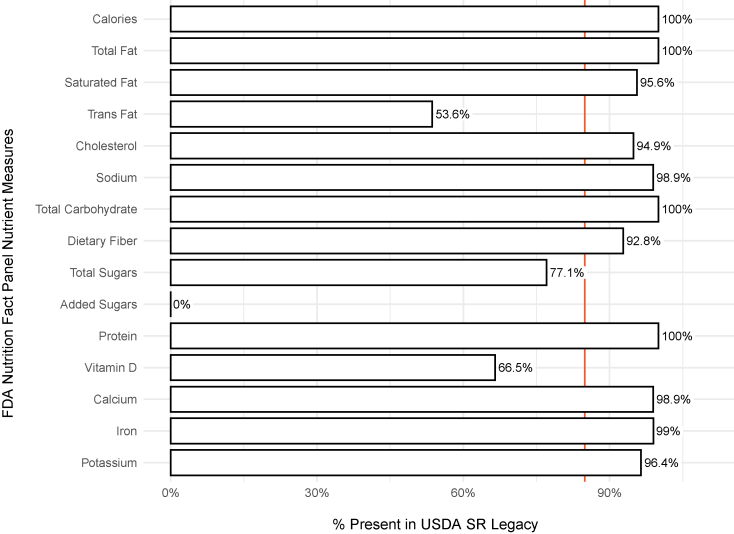
A summary of the FDA Nutrition Facts Panel nutrient measures available in SR Legacy. The presence of FDA-mandated NFP nutrient measures in SR Legacy food and nutrient data was used as an indicator of quality and completeness of the data set. The percentage shown for an NFP nutrient measure represents the percentage of foods in SR Legacy with content for that measure. The red vertical line indicates the mean percentage of all NFP nutrient measures reported in the SR Legacy. NFP, nutrition fact panel; SR, standard reference.

The completeness of NFP measures within SR Legacy were evaluated (Figure 3). The FDA regulates the nutrient measures required on the NFP label for all packaged foods sold in the United States. NFPs display 15 nutrient measures: calories, total fat, saturated fat, transfat, cholesterol, sodium, total carbohydrate, dietary fiber, total sugar, added sugar, protein, vitamin D, calcium, iron, and potassium [22]. Each value shown represents the percentage of all 7793 foods in SR Legacy for which content for a specific NFP nutrient measure was available. For example, as shown in Figure 3, all foods in SR Legacy (100%) reported a value for calories and total fat, but only roughly half reported a value for transfat (53.6%). SR Legacy data had a mean availability of 84.9% for NFP required nutrients (Figure 3, red vertical line). It should be noted that results shown in this study do not consider the date federal regulations were implemented such as requirements for labeling of added sugar or transfat.

**Figure 4:**
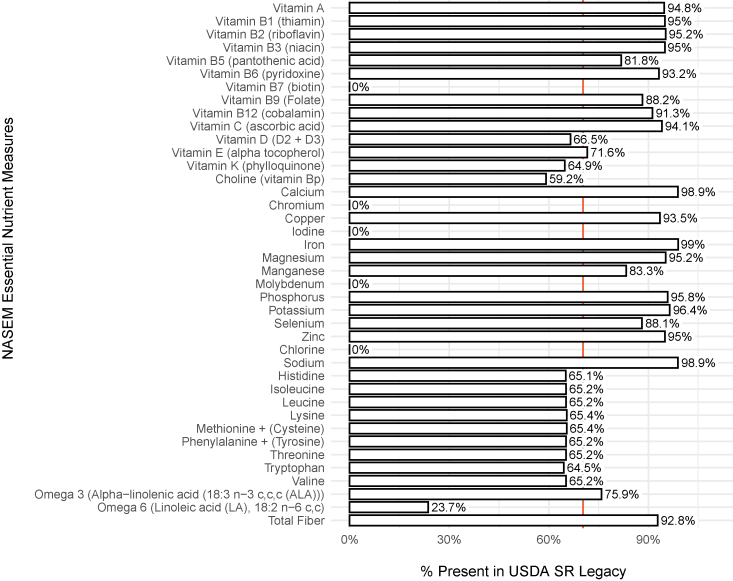
A summary of NASEM essential nutrient measures available in SR Legacy. The presence of NASEM essential nutrient measures in SR Legacy food and nutrient data were used as an indicator of quality and completeness of the data set. The percentage shown for an NASEM essential nutrient measure represents the percentage of foods in SR Legacy with recorded content for that measure. The red vertical line indicates the mean percentage of NASEM essential nutrient measures reported in SR Legacy. NASEM, National Academies of Sciences, Engineering, and Medicine; SR, standard reference.

The completeness of NASEM essential nutrient data represented in SR Legacy was also evaluated (Figure 4). These are the nutrients that cannot be synthesized by humans and must be consumed from foods or supplements; deficiency leads to a disease or death [23]. Each value shown represents the percentage of all 7793 foods in SR Legacy for which content for a specific NASEM essential nutrient measure was available. For example, 94.8% of foods in SR Legacy reported a value for vitamin A, whereas 66.5% reported a value for vitamin D (D2 + D3). As expected, SR Legacy entries were less likely to provide the broader range of NASEM essential nutrients, but the mean value of all NASEM nutrient availability was still 70.3% (Figure 4). It is worth noting that results shown in this study do not consider grouped laboratory analyses (for example, amino acid assay).

**Figure 5:**
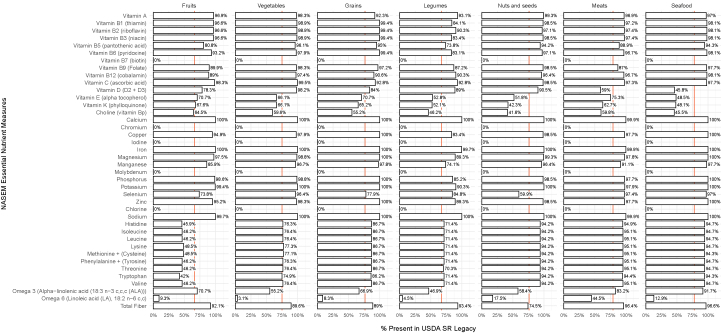
A summary of NASEM essential nutrient measures available in SR Legacy by the food group. SR Legacy data were analyzed for NASEM essential nutrient measures according to the food group based on the USDA MyPlate food groups. Percentages indicate the number of foods in each food grouping for which the content of each NASEM essential nutrient is provided in SR Legacy. The red vertical lines indicate the mean percentage in each food group. NASEM, National Academies of Sciences, Engineering, and Medicine; SR, standard reference.

Because some nutrients naturally occur in some foods and not others, we analyzed the availability of NASEM nutrient measures in SR Legacy based on the food groups (Figure 5). Food groups were those used in the USDA’s MyPlate. Overall, the nutrient measures available in each food group were fairly equivalent, with mean values of 66.3% for fruit, 74.9% for vegetables, 76.5% for grains, 67.5% for legumes, 76.3% for nuts and seeds, 79.2% for meats, and 77.8% for seafood.

**Figure 6:**
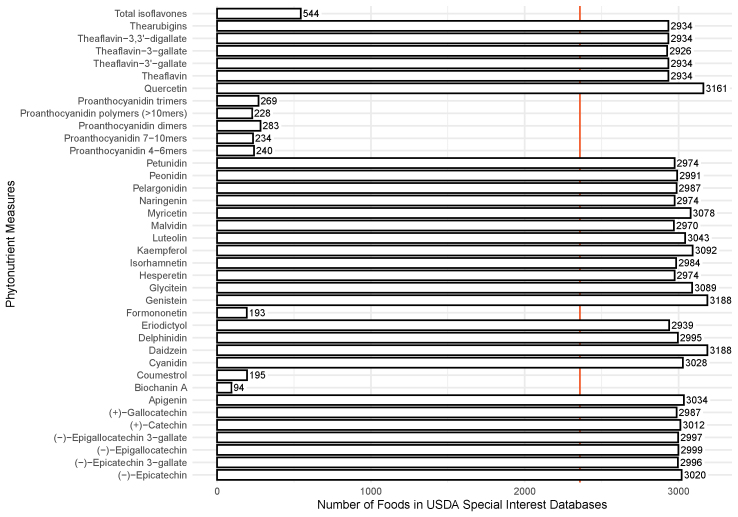
A summary of phytonutrient measures in the USDA Special Interest Databases. The 4 USDA Special Interest Databases relating to flavonoids, proanthocyanidins, and isoflavones were evaluated for phytonutrient data. The number shown for each phytonutrient measure represents the count of unique foods across all 4 Special Interest Databases for which the content of the specified phytonutrient measure is provided. The red vertical line indicates the mean number of foods for which phytonutrient data is provided.

The USDA currently provides the most comprehensive source of phytonutrient data. Therefore, phytonutrient measures were collected from the most current versions of the 4 USDA Special Interest Databases and were analyzed to quantify the quality of the available data for its completeness (Figure 6). In total, 38 phytonutrient measures were provided and analyzed across the unique foods of all 4 Special Interest Databases; however, the availability of each phytonutrient measure varied. For example, total isoflavone was provided for 544 foods, whereas quercetin was available for 3161 foods. It is important to clarify that foods in the USDA Special Interest Databases are unique to those databases: not all of them are represented in SR Legacy.

The date of addition or modification specifies when each nutrient measure value was added to the database or was last modified, this provides insight into the age and frequency of updates. Most nutrient measures have been updated. The date of addition or modification is provided for 99.8% of nutrient measures in SR Legacy, 0.2% of measures have no information on addition or modification. The oldest nutrient measures in SR Legacy were added in 1976, accounting for 1.0% of measures. The mean date of addition or modification was 5 June, 2003; however, 50% of measures were added after the median date of 1 May, 2006.

### Characterization of food and nutrient data sources

This review of food and nutrient data sources confirmed that the USDA databases predominated as the foundation of food and nutrient data used in studies worldwide and were incorporated into 57.7% of all sources reviewed. In other instances, the data source documentation indicated that “international sources” were included in their data, which were identified as likely containing the USDA data increasing the total to 84%. This review did not capture information on how nutrient data were used for the analysis or any limitations stated by the authors. The prevalence of the USDA food composition data affirms the use of SR Legacy in this review as a surrogate to assess the quality of nutrient data currently in use in the published studies.

**Figure 7:**
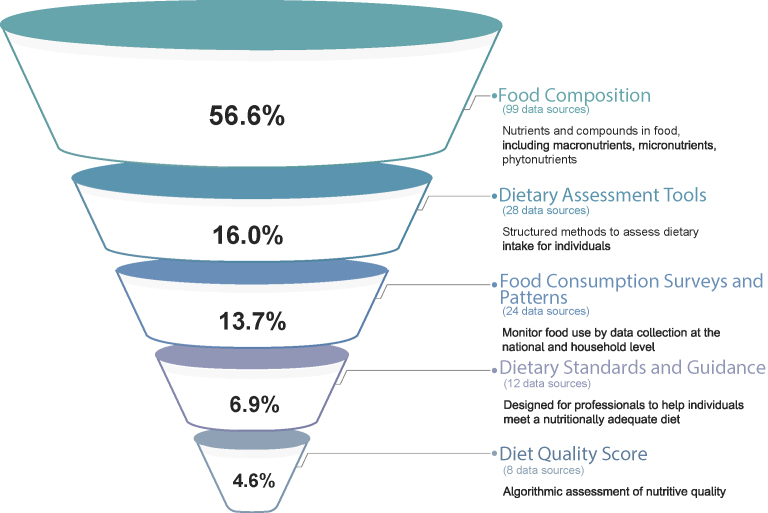
The food and nutrient data sources organized by purpose. The food and nutrient data sources listed in Supplemental Table 1 were organized into categories according to their purpose or primary use.

Supplemental Table 1 provides a full report of the 175 food and nutrient data sources collected according to the analysis as shown in Figure 2. Those food and nutrient data sources were organized into categories representing the major uses of the data (Figure 7). Among those sources, food composition data sources predominated (56.6%), followed by dietary assessment tools (16.0%), food consumption surveys and patterns (13.7%), dietary standards and guidance (6.9%), and diet quality score (4.6%). Sorting the food and nutrient data sources in this manner revealed a key relationship among all sources: all categories used food composition data in some way. Of the 175 food and nutrient data sources, 4 (2.2%) were not represented in Figure 7. Two did not fall into any major categories: the Diet-Related Fibers and Human Health Outcomes Database and Food Balance Sheets. Two were lacking sufficient information to be categorized: The Fred Hutchinson Cancer Research Center and the National Cancer Institute standard algorithm.

**Figure 8:**
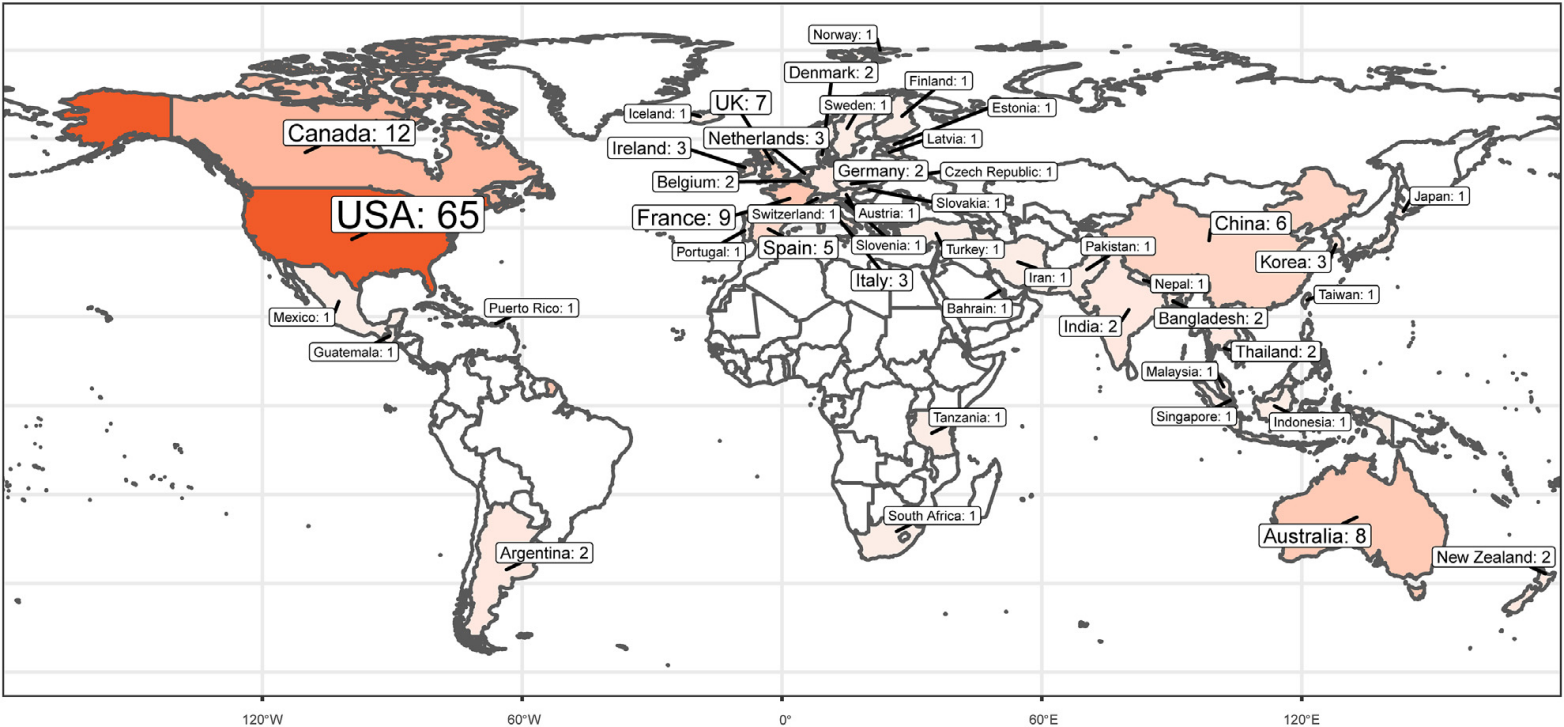
The food and nutrient data sources organized by the country of origin. The food and nutrient data sources from Supplemental Table 1 were characterized according to their respective countries of origin. The frequency of occurrence in a given country is depicted by the color intensity: countries in which the most sources originate appear in dark orange; countries in which fewer sources originate appear in lighter shades of orange. No data were identified for countries colored white. Numbers along the bottom of the map indicate the latitudes. Data sources created by multinational cooperatives were excluded from the figure.

Figure 8 shows a world map depicting the origins of the food and nutrient data sources in Supplemental Table 1 by the country. The data sources collected originated from 48 different countries and territories. Of all data types and sources collected, almost half (43.0%) originated in North America. Most of the data originated in the United States (37.1%) and Canada (6.9%). Another interesting finding was that food composition data was the most common food and nutrient data type throughout the world: 46 of the 48 countries represented had developed country-specific food composition data.

### FAIR principles: characterization of food and nutrient data

As described earlier, successfully implementing PN will require analyzing and integrating enormous amounts of data, and food and nutrient databases are foundational to this effort. The FAIR principles—findable, accessible, interoperable, and reusable—go beyond measuring data quality to interrogating how data are structured and used. This provides a deeper level of understanding and value for those who will be actively using food and nutrient data to create meaningful PN recommendations.

#### Findable

As described earlier, “findable” refers to the ease of locating a data source, usually by entering some of the available metadata into a search engine. The 175 data sources we collected included mentions of food and nutrient data in publications, publisher websites, and additional food and nutrient data sources (Supplemental Table 1 and Figure 2). Of those 175 data sources, only 154 (88.0%) were findable. For example, the Star of Nutrition software was referenced and used to track dietary intake. Despite a thorough web search, the Star of Nutrition source could not be found. Although our inability to find this missing source does not confirm its extinction, it does highlight the barriers to locating and replicating said research.

**Figure 9:**
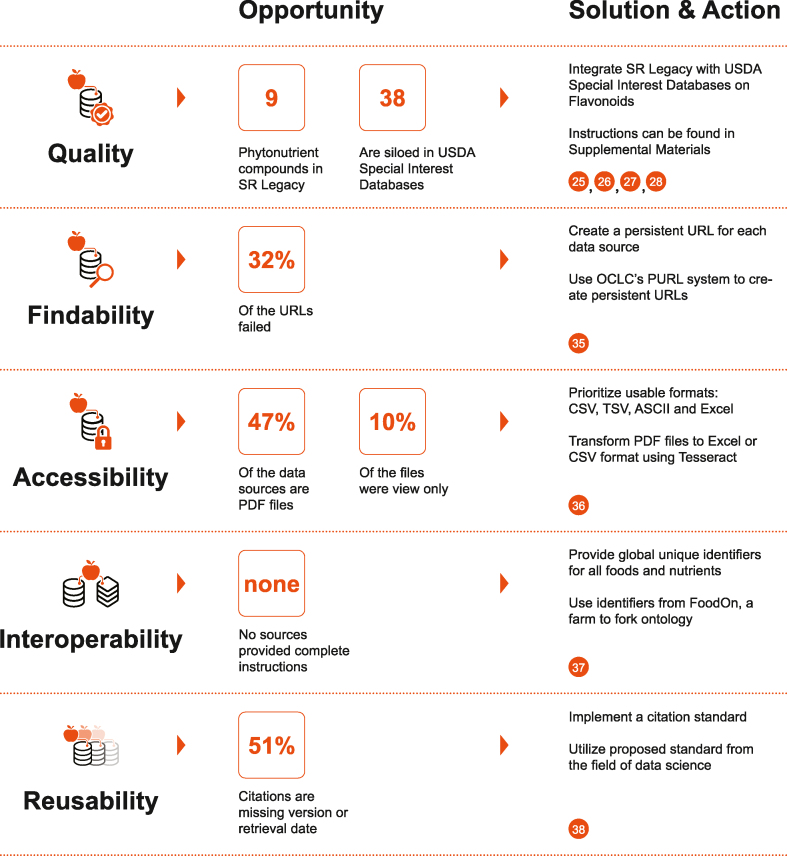
The opportunity analysis and solutions for improving data for precision nutrition. The center column of this matrix identifies current opportunities for improving food and nutrient data quality and FAIRness. The right-hand column lists corresponding solutions and actions that are simple and robust and would be easy to implement. Numerals in orange dots refer to corresponding citations [[25], [26], [27], [28],[35], [36], [37], [38]]. Instructions for database integration are detailed in the **Supplemental Material**. ASCII, American Standard Code for Information Interchange; CSV, comma-separated values; OCLC, the Online Computer Library Center; PURL, persistent uniform resource locator; SR, standard reference; TSV, tab-separated values.

In this review, 68.4% of URL links provided by citations worked at the time of collection. Most people have experienced the pain of a broken URL, when the user is sent to the dreaded “404 page not found” instead of the intended destination. The most findable data sources, data published in a scientific journal or the USDA data, for example, have unique persistent identifiers such as DOIs or static URLs. Although data sources were found using the URLs, the persistence of these URLs remains unknown. Such information must be created and provided by the data supplier. The problem of broken URLs can only be solved by adopting Persistent Uniform Resource Locators or implementing a handler system (Figure 9)

Other examples of less-findable data sources were those embedded in food composition data indexes such as the International Network of Food Data Systems (INFOODS) [30] and LanguaL [19], which are websites that provide links to available data sources or host copies of static sources. Such embedded data sources were not independently findable through a Google search. Instead, they could only be found by visually scanning a food composition data index webpage.

#### Accessible

As explained earlier, “accessible” refers to data that can be acquired. Of the 154 findable data sources, 78.6% (57/121 sources) could be accessed without payment or authorization. Of these sources, 43.8% were exportable in an Excel and/or CSV format, 11.6% were exportable in Microsoft Access and SAS formats, and 47.1% were exportable as PDFs. However, 9.7% (15 sources) of findable sources were viewable and searchable but were not exportable (Figure 9). Using data from those sources requires individually searching for each entry, then manually recording the entry into a usable format. For example, to use food composition data from the Spanish Food Composition Database (BEDCA), users must manually look up each food and record it into a spreadsheet—a time-intensive and potentially error-prone process.

The problem of data trapped in PDF or image formats can be resolved using optical character recognition (OCR), which is the electronic conversion of images into machine-encoded text, which is widely used as a form of data entry. However, OCR does occasionally misread characters, thus leaving open the possibility for errors to be incorporated into data and downstream analyses.

#### Interoperable

Interoperability, in this context, is defined by the ability to harmonize or connect multiple sources into a usable network, typically relying on linking unique identifiers. This feature allows for easier access to data from disparate sources.

The USDA databases are the largest currently available globally connected network of food and nutrient data, with a network of identifiers connecting NHANES, the What We Eat in America database, FNDDS, SR Legacy, FDC, the Dietary Supplement Label Database, the USDA Special Interest Databases on Flavonoids, and many others. Although these databases have a high level of interoperability, it can be challenging for users to identify how they integrate. There is no official guide to joining them and identifier names can vary between databases.

At present, there is no way to fully identify the true state of interoperability within the food and nutrient data landscape owing to a lack of documentation and instruction on the available linkages of food and nutrient data sources (Figure 9). Currently, common variables that can be used to connect databases must be identified through trial and error. For example, documentation in FNDDS claims that FNDDS and NHANES can be integrated. To find this connection, both databases must be extracted and examined for similar variables to conclude that the NHANES variable “USDA Food Code” (FDCD) can be joined to the FNDDS variable “Food Code” (food_code). Interoperability will be most facilitated by a common index such as those implemented in food ontologies.

#### Reusable

As described earlier, “reusable” refers to data that have a quality metadata available to enable repeated and reliable access. An example of highly reusable data is the FDC food composition data because the FDC provides the title, a persistent URL, the version through a publication date, and an FDC ID to identify each food. Paradoxically, the USDA suggested citation for FDC itself, found on the main FDC website, is “U.S. Department of Agriculture, Agricultural Research Service. FDC, 2019. fdc.nal.usda.gov.” Because this citation does not include the version or the retrieval date, there would be no way to pinpoint what data were used.

Of the food composition data category, 48.9% of citations provided metadata on either the version or the retrieval date, whereas 51.1% (70/137 citations) did not (Figure 9). Currently, there is no standard for the citation of food composition data in the nutritional sciences. Acceptance and implementation of a citation standard will be necessary to assure authors provide enough information for the data they use in research to be identified and reused.

## Discussion and Perspectives

An essential question for nutrition science is the question of what needs to be performed to make PN truly robust. As illustrated in Figure 1, food composition data inform all aspects of PN. However, nutrition science does not currently have established standards for defining and ensuring the quality of that data. This study began by determining the current state of the field regarding food and nutrient data quality. To establish a minimum starting point, “completeness” was defined in a database as the inclusion of data for all 15 NFP nutrient measures and all 40 NASEM essential nutrient measures, recorded for each food. The gold standard USDA SR Legacy database was not complete for either NFP nutrient measures (mean value of 84.9%) (Figure 3) or NASEM essential nutrient measures (mean value of 70.3%) (Figure 4). Because phytonutrients are also important for health, we examined the availability of 38 phytonutrient measures among the unique foods in the 4 USDA Special Interest Databases (Figure 6). With an estimated 50,000 phytonutrients, those 38 represent only a tiny fraction of the possible food composition data to be gathered.

As far as is known, these findings represent the most comprehensive analysis of the completeness of currently used food and nutrient databases. The foundational measure of comprehensiveness in nutrient data should be the inclusion of all essential nutrients, defined as the NFP measurements and NASEM essential nutrients, and a comprehensive inclusion of phytonutrients.

To further describe the landscape of food composition data sources, food and nutrient data sources were characterized in publications from the most widely read nutrition journals. We identified 97 publications and ultimately collected a set of 175 data sources from around the world (Figure 2 and Supplemental Table 1). We found that the USDA databases were the predominant source of food and nutrient data that were incorporated into more than half (57.7%) of our collected data sources. Moreover, by organizing the set of data sources according to the major uses of their data, it was found that each use-defined category relied on food composition data (Figure 7). As shown in Figure 7, data sources are funneled from broader categories to more specialized ones, with each category depending on the preceding less specialized database. Diet quality scores depend on dietary standards, which are shaped by food consumption surveys and dietary patterns, which in turn rely on dietary assessment tools informed by food composition data. This observation provides insight into the frequency of use, need, and the areas of opportunity for improvement.

The matrix in Figure 9 summarizes the findings regarding the FAIRness of food composition data, identifying both the key opportunities (how much the status quo falls short of FAIRness) and the most practical corresponding solutions.

Focusing on interoperability will foster the greatest overall improvement in the database quality. The analysis in this review indicates that all currently available data sources are deficient in some ways. Therefore, the ability to link data sources will allow users to integrate complementary information from different sources to create a more comprehensive set of measures. This approach will allow researchers to use existing data to establish a definitive understanding of what is currently known and what is missing. Once existing data have been integrated, researchers can then improve data quality based on precision and accuracy. But if data are left soiled as it is at the present time, precious time and dollars will be wasted.

Making food composition data more complete and more FAIR will provide critical information for the implementation of PN, which can in turn have a profound positive effect on human health. An example of the potential effect of such databases on consumers is the popularity of applications, such as MyFitnessPal, which relies on publicly available food composition data, USDA, and others. This platform has over 100 million downloads and is the second top grossing health and fitness app on the Google Play store as of January 2023 [31,32].

The intention of this strategic approach is to provide an actionable set of plans that support both data suppliers and scientists alike with the ultimate goal to serve consumers. To accomplish that objective, an appropriate call to action is as follows: *1*) nutrition scientists should actively collaborate with data science experts to ensure that food and nutrient data can work with the ever-expanding associated scientific disciplines; *2*) organizations such as the American Society for Nutrition should continue to promote PN as an important emerging nutrition topic and advocate for high quality and complete food composition data that follows FAIR principles [33]; and *3*) ambitious research, such as the NIH Nutrition for Precision health study should set high-quality standards for food composition data that are implemented across the clinical study sites.

Current dietary recommendations, such as the Dietary Reference Intakes, are designed to prevent deficiency and reduce risk of noncommunicable diseases. In high-income Western societies, another goal of dietary recommendations has been developed to address chronic diseases [34]. PN provides an opportunity to transition away from a disease-focused model to an individualized one of the optimal personal health. With PN in its infancy, this is the ideal time for nutrition scientists to recognize and elevate the importance of food and nutrient data and in doing so to set the bar high. The ambitious, comprehensive PN approach will be best positioned to develop effective diet interventions to achieve optimal health if nutrition science focuses on ensuring that food composition data are complete and FAIR.
