# Supplementary material for: Perspective: A Comprehensive Evaluation of Data Quality in Nutrient Databases
Source: Adv Nutr. 2023 Feb 25;14(3):379–91. doi: 10.1016/j.advnut.2023.02.005 (PMC10201679; doi:10.1016/j.advnut.2023.02.005)
Supplement: Multimedia component 1 [file mmc1.docx]

**Online Supplementary Table 1.** Summary of food and nutrient data sources^^[[1]](#footnote-0)^^

| Data Source | Data Category | USDA Inclusion | Format | Export File Type | Accessibility | Country of Origin |
| --- | --- | --- | --- | --- | --- | --- |
| Analysis of theaflavins and thearubigins from black tea extract by MALDI-TOF mass spectrometry | Food Composition | no | Downloadable Publication, Purchase Required | PDF | Restricted Access | USA |
| Australian Food, Supplement and Nutrient Database (AUSNUT) | Food Composition | yes | Downloadable Tables | MS Excel | Open Access | Australia |
| Canadian Nutrient File (CNF) | Food Composition | yes | Online Access and Downloadable Tables | MS Access, MS^^[[2]](#footnote-1)^^ Excel, CSV | Open Access | Canada |
| Carotenoids: Alpha-Carotene, Beta-Carotene, Beta-Cryptoxanthin, Lycopene, Lutein and Zeaxanthin | Food Composition | yes | Downloadable Tables | PDF | Open Access | USA |
| Chinese Food Composition Tables | Food Composition | unknown | Book | None | Restricted Access | China |
| Comparison of nutritional and chemical parameters of soymilk and cow milk | Food Composition | no | Downloadable Publication, No Purchase Required | PDF | Open Access | Iran |
| Computerized Heterocyclic Amines Resource for Research in Epidemiology of Disease | Food Composition | unknown | Software, Access must be requested via email | SAS | Restricted Access | USA |
| HPB Food Composition System Singapore (FOCOS) | Food Composition | yes | Online Access and Book | Viewable in application only | Open Access | Singapore |
| Energy drinks: an assessment of their market size, consumer demographics, ingredient profile, functionality, and regulations in the United States | Food Composition | no | Downloadable Publication, No Purchase Required | PDF | Open Access | USA |
| Eurostat | Food Composition | no | Online Access and Downloadable Tables | TSV, SDMX | Open Access | EU |
| Fineli | Food Composition | likely | Online Access and Downloadable Tables | MS Excel, CSV | Open Access | Finland |
| Food and Nutrient Database for Dietary Studies (FNDDS) | Food Composition | yes | Downloadable Tables | SAS, MS Access, MS Excel, CSV, JSON | Open Access | USA |
| Food chain approach to lowering the saturated fat of milk and dairy products | Food Composition | no | Downloadable Publication, Purchase Required | PDF | Restricted Access | UK |
| Food Label Information Program (FLIP) | Food Composition | no | Not publicly available | Unknown | Inaccessible | Canada |
| Frida Food Data | Food Composition | yes | Online Access and Downloadable Tables | MS Excel | Open Access | Denmark |
| General Mills Bell Institute of Health & Nutrition (BIHN) Whole Grain Database | Food Composition | unknown | Not publicly available | Unknown | Inaccessible | USA |
| Harvard T.H. Chan School of Public Health Nutrition Department's Food Composition Table | Food Composition | unknown | Downloadable Tables | Unknown | Open Access | USA |
| Hong Kong food-composition table | Food Composition | unknown | Online Access and Downloadable Tables | Unknown | Open Access | China |
| HPLC determination of catechins and caffeine in tea. Differentiation of green, black and instant teas | Food Composition | no | Downloadable Publication, Purchase Required | PDF | Restricted Access | Spain |
| Indian Food Composition Tables | Food Composition | no | Downloadable Tables | PDF | Open Access | India |
| International Network of Food Data Systems (INFOODS) | Food Composition | likely | Downloadable Tables | PDF, MS Excel | Open Access | Global |
| Irish National Food Ingredient Database | Food Composition | unknown | No longer in use | Unknown | Inaccessible | Ireland |
| Korean Standard Food Composition Table | Food Composition | unknown | Online Access and Downloadable Tables | PDF | Open Access | Korea |
| McCance and Widdowson’s The Composition of Foods | Food Composition | unknown | Book | PDF | Restricted Access | UK |
| Nutrient composition, protein quality and antinutritional factors of some varieties of dry beans (Phaseolus vulgaris) grown in Burundi | Food Composition | no | Downloadable Publication, Purchase Required | PDF | Restricted Access | Canada |
| Polyamine contents in current foods: a basis for polyamine reduced diet and a study of its long term observance and tolerance in prostate carcinoma patients | Food Composition | no | Downloadable Publication, Purchase Required | PDF | Restricted Access | France |
| Sistema de Evaluación de Hábitos Nutricionales y Consumo de Nutrimentos (SNUT) | Food Composition | yes | Downloadable Tables | PDF | Open Access | Mexico |
| SMILING (Sustainable Micronutrient Interventions to Control Deficiencies and Improve Nutritional Status and General Health in Asia) | Food Composition | yes | Downloadable Tables | MS Excel | Open Access | France |
| Spanish Food Composition Tables | Food Composition | yes | Online Access | CD-ROM | Open Access | Spain |
| Standard Tables of Food Composition in Japan | Food Composition | unknown | Downloadable Tables | PDF, MS Excel | Open Access | Japan |
| Sugar content of popular sweetened beverages based on objective laboratory analysis: focus on fructose content | Food Composition | no | "Downloadable Publication, Purchase Required" | PDF | Restricted Access | USA |
| Tabla de Composición de Alimentos de Centro América | Food Composition | yes | Downloadable Tables | PDF | Open Access | Guatemala |
| Table of Taiwan Food Composition | Food Composition | unknown | unknown | Unknown | Inaccessible | Taiwan |
| Tanzania Food Composition Tables | Food Composition | yes | Downloadable Tables | PDF, MS Excel | Open Access | Tanzania |
| The Australian Food Composition Database (NUTTAB) | Food Composition | yes | Online Access and Downloadable Tables | MS Excel | Open Access | Australia |
| The Dutch Food Composition Database (NEVO) | Food Composition | yes | Online Access and Downloadable Tables | MS Excel, CSV | Open Access | Netherlands |
| The German Nutrient Database (BLS) | Food Composition | unknown | Online Access, Purchase required | Unknown | Restricted Access | Germany |
| The Spanish Food Composition Database (BEDCA) | Food Composition | yes | Online Access | Viewable in application only | Open Access | Spain |
| Türkomp | Food Composition | no | Online Access | Viewable in application only | Open Access | Turkey |
| USDA Branded Food Products Database | Food Composition | yes | Online Access and Downloadable Tables | CSV, MS Access, JSON | Open Access | USA |
| USDA Database for the Flavonoid Content of Selected Foods | Food Composition | yes | Downloadable Tables | MS Access, PDF | Open Access | USA |
| USDA Database for the Isoflavone Content of Selected Foods | Food Composition | yes | Downloadable Tables | MS Access, PDF | Open Access | USA |
| USDA Database for the Proanthocyanidin Content of Selected Foods | Food Composition | yes | Downloadable Tables | MS Access | Open Access | USA |
| USDA Food Data Central | Food Composition | yes | Online Access and Downloadable Tables | CSV, MS Access, JSON | Open Access | USA |
| USDA National Fluoride Database of Selected Beverages and Foods | Food Composition | yes | Online Access and Downloadable Tables | PDF, MS Excel | Open Access | USA |
| USDA National Nutrient Database for Standard Reference | Food Composition | yes | Downloadable Tables | CSV, MS Access, JSON | Open Access | USA |
| Variations in caffeine and chlorogenic acid contents of coffees: what are we drinking? | Food Composition | no | Downloadable Publication, No Purchase Required | PDF | Open Access | UK |
| World Food Dietary Assessment System | Food Composition | yes | Software, No longer in use | Unknown | Inaccessible | USA |
| Composition of foods: Raw, processed, prepared (Agriculture handbook) | Food Composition | yes | Book, Free Downloadable PDF | PDF | Open Access | USA |
| AusFoods | Food Composition | yes | Purchase required | Unknown | Restricted Access | Australia |
| Composition of foods integrated dataset (CoFID) | Food Composition | yes | Downloadable Tables | MS Excel | Open Access | UK |
| Indonesian Food Composition Table | Food Composition | likely | Online Access | PDF | Open Access | Indonesia |
| Phenol-Explorer | Food Composition | yes | Online Access and Downloadable Tables | MS Excel, CSV | Open Access | France |
| Portie-online | Food Composition | unknown | Online Access and Downloadable Tables | CSV | Open Access | Netherlands |
| Nutrient Information Inquiry System (NIIS) | Food Composition | yes | Online Access | Viewable in application only | Open Access | China |
| Swiss Food Composition Database | Food Composition | no | Online Access and Downloadable Tables | MS Excel | Open Access | Switzerland |
| FooDB | Food Composition | yes | Online Access and Downloadable Tables | CSV, XML, JSON, MySQL Dump file | Open Access | Canada |
| Swedish Food Agency food database | Food Composition | no | Online Access and Downloadable Tables | MS Excel | Open Access | Sweden |
| FoodEXplorer (EuroFIR) | Food Composition | yes | Online Access and Downloadable Tables, Purchase Required | food data transport package (FDTP), MS Excel | Restricted Access | EU |
| Icelandic database on the chemical content of food (ÍSGEM) | Food Composition | yes | Online Access and Downloadable Tables | PDF | Open Access | Iceland |
| "Austrian Food Composition Database (ÖNWT, Österreichische Nährwerttabelle)" | Food Composition | yes | Online Access and Downloadable Tables | RTF | Open Access | Austria |
| Czech Food Composition Database | Food Composition | yes | Online Access and Downloadable Tables, Requires Registration | MS Excel | Open Access | Czech Republic |
| NutriData food composition database | Food Composition | yes | Online Access and Downloadable Tables | MS Excel | Open Access | Estonia |
| Ciqual French food composition table | Food Composition | yes | Online Access and Downloadable Tables | MS Excel, XML | Open Access | France |
| New Zealand Food Composition Database | Food Composition | yes | Online Access and Downloadable Tables | ASCII TXT, MS Excel | Open Access | New Zealand |
| Latvia's national food composition database | Food Composition | likely | unknown | Unknown | Inaccessible | Latvia |
| Food Composition Database for Epidemiological Studies in Italy (Banca Dati di Composizione degli Alimenti per Studi Epidemiologici in Italia – BDA) | Food Composition | yes | Online Access and Downloadable Tables | MS Excel | Open Access | Italy |
| Slovak Food Composition Data Bank (SFCDB) | Food Composition | yes | Online Access | Viewable in application only | Open Access | Slovakia |
| Portuguese Food Composition Database | Food Composition | unknown | Online Access and Downloadable Tables | MS Excel | Open Access | Portugal |
| Norwegian Food Composition Table | Food Composition | yes | Online Access and Downloadable Tables | OpenOffice, MS Excel | Open Access | Norway |
| Dr. Duke's Phytochemical and Ethnobotanical Databases | Food Composition | yes | Online Access and Downloadable Tables | PDF, MS Excel, CSV | Open Access | USA |
| Malaysian Food Composition Database (MyFCD) | Food Composition | yes | Online Access | Viewable in application only | Open Access | Malaysia |
| Food Composition Table for Nepal | Food Composition | no | Downloadable Tables | PDF | Open Access | Nepal |
| Food Composition Table for Pakistan | Food Composition | yes | Downloadable Tables | PDF | Open Access | Pakistan |
| Thai Food Composition Database | Food Composition | no | Online Access and Downloadable Tables | PDF, MS Excel | Open Access | Thailand |
| FAO/INFOODS Food Composition Table for Western Africa (WAFCT 2019) | Food Composition | yes | Downloadable Tables | PDF, MS Excel | Open Access | Italy |
| Global Food Matters Database | Food Composition | yes | Not publicly available | Unknown | Restricted Access | USA |
| Food Safety Collaborative Platform (FOSCOLLAB) | Food Composition | likely | Online Access and Downloadable Tables | MS Excel, CSV | Open Access | Global |
| FlavorDB | Food Composition | yes | Online Access and Downloadable Tables | Mol2, 2D Image, SDF, JSON | Open Access | India |
| ASEAN Food Composition Database | Food Composition | unknown | Requires registration to access | PDF | Open Access | Thailand |
| Crop Composition Database (CCDB) | Food Composition | yes | Requires registration to access | HTML, PDF, CSV | Open Access | USA |
| South African Food Data System (SAFOODS) | Food Composition | yes | Online Access | Viewable in application only | Open Access | South Africa |
| Fred Hutch Glycemic Index Database | Food Composition | yes | Not publicly available | Unknown | Restricted Access | USA |
| Korean Flavanoid and Phenolic acid Database | Food Composition | likely | Online Access and Downloadable Tables | MS Excel | Open Access | Korea |
| ARGENFOODS | Food Composition | no | Online Access and Downloadable Tables | PDF, MS Excel | Open Access | Argentina |
| DHQ Nutrient and Food Group Database | Food Composition | yes | Downloadable Tables | CSV | Open Access | USA |
| Food Composition Tables for Kingdom of Bahrain | Food Composition | no | Downloadable Tables | PDF | Open Access | Bahrain |
| A Food Composition Database for Bangladesh with Special reference to Selected Ethnic Foods | Food Composition | no | Downloadable Tables | PDF | Open Access | Bangladesh |
| Food Composition Table for Bangladesh | Food Composition | yes | Downloadable Tables | PDF, MS Excel | Open Access | Bangladesh |
| NUBEL | Food Composition | yes | Downloadable Tables, Purchase required | PDF | Restricted Access | Belgium |
| INTERNUBEL | Food Composition | yes | Online Access | Viewable in application only | Open Access | Belgium |
| ARFenol-Foods | Food Composition | no | Online Access and Downloadable Tables, Requires Registration | PDF | Open Access | Argentina |
| Pacific Nutrient Database (PNDB) | Food Composition | yes | Online Access and Downloadable Tables | MS Excel, Stata, R, Python, PowerBI | Open Access | France |
| FDA Total Diet Study (TDS) | Food Composition | yes | Downloadable Tables | CSV, PDF | Open Access | USA |
| Nutritionix | Food Composition | likely | Online Access and Downloadable Tables | CSV | Open Access | USA |
| P-MetDB | Food Composition | yes | Not publicly available | Unknown | Restricted Access | USA |
| MetaboFood-KDB | Food Composition | yes | Online Access | Viewable in application only | Open Access | USA |
| PhytoHub | Food Composition | yes | Online Access and Downloadable Tables | CSV | Open Access | France |
| Database of Flavonoid Values for USDA Food Codes | Food Composition | yes | Downloadable Tables | SAS, MS Access, PDF, TXT | Open Access | USA |
| Automated Self-Administered 24-Hour (ASA24®) Dietary Assessment Tool | Dietary Assessment Tools | yes | Online Access | MS Excel, CSV | Open Access | USA |
| Cronometer Pro | Dietary Assessment Tools | yes | Software, Purchase required | Unknown | Restricted Access | Canada |
| Diet*Calc Analysis Software | Dietary Assessment Tools | yes | Software, No Purchase Required | TXT, Windows EXE | Open Access | USA |
| NCI Dietary Analysis System (Dietsys) | Dietary Assessment Tools | likely | Software, No longer in use | Diskette | Inaccessible | USA |
| ESHA’s Food Processor® Nutrition Analysis | Dietary Assessment Tools | yes | Software, Purchase required | RTF, PDF, CSV, MS Excel | Restricted Access | USA |
| FoodFlip© | Dietary Assessment Tools | no | Mobile App, Not publicly available | Viewable in application only | Inaccessible | Canada |
| FoodSMART | Dietary Assessment Tools | unknown | Mobile App | Viewable in application only | Open Access | USA |
| FoodSwitch | Dietary Assessment Tools | yes | Mobile App | Viewable in application only | Open Access | Australia |
| FoodWorks | Dietary Assessment Tools | yes | Software, Purchase required | Windows EXE, SAS, SPSS, MS Excel | Restricted Access | Australia |
| MyNutriCart | Dietary Assessment Tools | yes | Mobile App | Viewable in application only | Open Access | Puerto Rico |
| Fruit & Vegetable Screeners in the Eating at America's Table Study (EATS) | Dietary Assessment Tools | yes | Downloadable Tables | PDF | Open Access | USA |
| Nutridash | Dietary Assessment Tools | yes | Requires registration to access | Unknown | Restricted Access | USA |
| Nutrilet | Dietary Assessment Tools | unknown | Software, unknown | Unknown | Inaccessible | Spain |
| Nutritics | Dietary Assessment Tools | yes | Software, Purchase required | MS Excel, PDF | Restricted Access | Ireland |
| Nutrition Data System for Research (NDSR) | Dietary Assessment Tools | yes | Software, Purchase required | zipped TXT | Restricted Access | USA |
| NutritionQuest | Dietary Assessment Tools | likely | Software, Purchase required | Unknown | Restricted Access | USA |
| Oxford WebQ | Dietary Assessment Tools | yes | Online Access, Access must be requested via email | Unknown | Restricted Access | UK |
| PCN Pro 1.0© | Dietary Assessment Tools | unknown | Not publicly available | Unknown | Inaccessible | Spain |
| SmartAPPetite | Dietary Assessment Tools | likely | Mobile App | Viewable in application only | Open Access | Canada |
| Star of Nutrition software | Dietary Assessment Tools | unknown | unknown | Unknown | Inaccessible | China |
| EaTracker | Dietary Assessment Tools | likely | No longer in use | Unknown | Inaccessible | Canada |
| MyFitnessPal | Dietary Assessment Tools | yes | Mobile App | Viewable in application only | Open Access | USA |
| Nutri-Score label | Dietary Assessment Tools | likely | Mobile App | PDF | Open Access | France |
| SCREEN © | Dietary Assessment Tools | yes | Online Access | PDF | Open Access | Canada |
| Open Platform for Clinical Nutrition (OPKP) | Dietary Assessment Tools | yes | Requires registration to access | MS Excel, PDF | Open Access | Slovenia |
| FAOSTAT | Dietary Assessment Tools | yes | Online Access and Downloadable Tables | PDF, CSV, JSON, XML | Open Access | Global |
| INDDEX24 Dietary Assessment Platform | Dietary Assessment Tools | unknown | Not publicly available | Unknown | Restricted Access | USA |
| SuperTracker | Dietary Assessment Tools | yes | No longer in use | MS Excel, JSON | Open Access | USA |
| China Health and Nutrition Survey (CHNS) | Food Consumption Surveys and Patterns | no | Requires registration to access | SAS | Restricted Access | China |
| Dutch National Food Consumption Survey (DNFCS) | Food Consumption Surveys and Patterns | yes | Online Access and Downloadable Tables | CSV, MS Excel | Open Access | Netherlands |
| EFSA Comprehensive European Food Consumption Database | Food Consumption Surveys and Patterns | likely | Downloadable Tables | CSV, MS Excel, PDF | Open Access | EU |
| Harvard Willett Food Frequency Questionnaire | Food Consumption Surveys and Patterns | likely | FFQ, Purchase required | PDF | Restricted Access | USA |
| Health ABC | Food Consumption Surveys and Patterns | no | Downloadable Tables | PDF | Open Access | USA |
| Health Professionals Follow-up Study (HPFS) | Food Consumption Surveys and Patterns | likely | Not publicly available | Unknown | Inaccessible | USA |
| Men's Lifestyle Validation Study (MLVS) | Food Consumption Surveys and Patterns | unknown | Not publicly available | Unknown | Inaccessible | USA |
| Nutrition Assessment shared resource (NASR) Food Frequency Questionnare | Food Consumption Surveys and Patterns | yes | FFQ, Purchase required | PDF | Restricted Access | USA |
| National Eating Trends® (NET®) | Food Consumption Surveys and Patterns | unknown | "Software, Purchase required" | Unknown | Restricted Access | USA |
| National Health and Nutrition Examination Survey (NHANES) | Food Consumption Surveys and Patterns | yes | Downloadable Tables | SAS | Open Access | USA |
| Nurses’ Health Study (NHS) | Food Consumption Surveys and Patterns | likely | Downloadable Tables | Unknown | Open Access | USA |
| NutriNet-Santé | Food Consumption Surveys and Patterns | unknown | unknown | Unknown | Inaccessible | France |
| The Continuing Survey of Food Intake by Individuals | Food Consumption Surveys and Patterns | yes | Downloadable Tables | SAS | Open Access | USA |
| What We Eat in America (WWEIA) | Food Consumption Surveys and Patterns | yes | Downloadable Tables | PDF | Open Access | USA |
| Women’s Health Initiative (WHI) Food Frequency Questionnaire (FFQ) | Food Consumption Surveys and Patterns | no | Downloadable Tables | PDF | Open Access | USA |
| The National Diet and Nutrition Survey (NDNS) | Food Consumption Surveys and Patterns | yes | Downloadable Tables | PDF | Open Access | UK |
| Canadian Community Health Survey (CCHS) | Food Consumption Surveys and Patterns | likely | Online Access and Downloadable Tables | MS Excel, CSV, TXT, JSON | Open Access | Canada |
| The German National Nutrition Survey (NVS) | Food Consumption Surveys and Patterns | unknown | unknown | Unknown | Inaccessible | Germany |
| Diet and Health Knowledge Survey (DHKS) | Food Consumption Surveys and Patterns | yes | No longer in use | PDF | Inaccessible | USA |
| FAO/WHO Global Individual Food consumption data Tool (GIFT) | Food Consumption Surveys and Patterns | yes | Online Access and Downloadable Tables | MS Excel | Open Access | Italy |
| National Teens' Food Survey (NTFS) and National Children's Food Survey (NCFS) | Food Consumption Surveys and Patterns | unknown | Downloadable Tables | PDF | Open Access | Ireland |
| Australian Health Survey (AHS) | Food Consumption Surveys and Patterns | yes | Online Access and Downloadable Tables | PDF, MS Excel | Open Access | Australia |
| Diet History Questionnaire (DHQ) | Food Consumption Surveys and Patterns | yes | Downloadable Tables | PDF, MS Word | Open Access | USA |
| Korean National Health and Nutrition Examination Survey (KNHANES) | Food Consumption Surveys and Patterns | unknown | unknown | PDF | Inaccessible | Korea |
| Australian Dietary Guidelines | Dietary Standards and Guidance | yes | Downloadable Tables | PDF | Open Access | Australia |
| Australian Guide to Healthy Eating | Dietary Standards and Guidance | yes | Downloadable Tables | PDF | Open Access | Australia |
| Dietary Guidelines for Americans | Dietary Standards and Guidance | yes | Downloadable Tables | PDF | Open Access | USA |
| Dietary Reference Intakes for Chinese | Dietary Standards and Guidance | no | Downloadable Tables | PDF | Open Access | China |
| Pyramid Servings Database (PSDB) | Dietary Standards and Guidance | yes | Downloadable Tables | SAS | Open Access | USA |
| USDA Food Patterns | Dietary Standards and Guidance | yes | Downloadable Tables | PDF | Open Access | USA |
| USDA Food Patterns Equivalents Database (FPED) | Dietary Standards and Guidance | yes | Downloadable Tables | MS Excel, MS Access, SAS | Open Access | USA |
| MyPyramid Equivalents Food Database (MPED) | Dietary Standards and Guidance | yes | Downloadable Tables | SAS, MS Access, CSV | Open Access | USA |
| USDA Food Guidance System (MyPlate) | Dietary Standards and Guidance | yes | Online Access and Downloadable Tables | PDF | Open Access | USA |
| Canada’s Food Guide | Dietary Standards and Guidance | unknown | Online Access and Downloadable Tables | PDF | Open Access | Canada |
| MyPyramid | Dietary Standards and Guidance | yes | No longer in use | PDF | Inaccessible | USA |
| Nordic Nutrition Recommendations (NNR) | Dietary Standards and Guidance | yes | Downloadable Tables | PDF | Open Access | Denmark |
| Alternative Healthy Eating Index (AHEI) | Diet Quality Score | yes | Downloadable Publication, No Purchase Required | PDF | Open Access | USA |
| Digestible Indispensable Amino Acid Score (DIAAS) | Diet Quality Score | no | Downloadable Publication, No Purchase Required | PDF | Open Access | New Zealand |
| Healthy Eating Index (HEI) | Diet Quality Score | yes | Downloadable Tables | PDF | Open Access | USA |
| SAIN,LIM | Diet Quality Score | yes | Downloadable Publication, No Purchase Required | PDF | Restricted Access | Canada |
| Nutrient-Rich Food (NRF) index | Diet Quality Score | yes | Downloadable Publication, Purchase Required | PDF | Restricted Access | USA |
| The protein digestibility-corrected amino acid score (PDCAAS) | Diet Quality Score | no | Downloadable Tables | PDF | Open Access | Global |
| SENS (Système d’Etiquetage Nutritionnel Simplifié) | Diet Quality Score | unknown | No longer in use | PDF | Inaccessible | France |
| Multiple Traffic Lights (MTL) | Diet Quality Score | yes | unknown | Unknown | Inaccessible | UK |
| Diet-Related Fibers and Human Health Outcomes Database | Other | yes | Online Access | MS Excel | Open Access | USA |
| Food Balance Sheets (FBS) | Other | yes | Online Access and Downloadable Tables | PDF | Open Access | Global |
| The Fred Hutchinson Cancer Research Center | Other | N/A^^[[3]](#footnote-2)^^ | Research Center | Not Applicable | Restricted Access | USA |
| The National Cancer Institute Standard Algorithm | Other | N/A | Research Center | Not Applicable | Restricted Access | USA |

1. ^Data was collected from May 28, 2021 to August 31, 2022^ [↑](#footnote-ref-0)
2. ^MS, Microsoft^ [↑](#footnote-ref-1)
3. ^N/A, Not Applicable^ [↑](#footnote-ref-2)
