# Supplementary material for: Perspective: A Comprehensive Evaluation of Data Quality in Nutrient Databases
Source: Adv Nutr. 2023 Feb 25;14(3):379–91. doi: 10.1016/j.advnut.2023.02.005 (PMC10201679; doi:10.1016/j.advnut.2023.02.005)
Supplement: Multimedia component 2 [file mmc2.docx]

**Online Supplemental Material**

**Integration of USDA Special Interest Databases with SR Legacy**

This document serves as a guide for integrating USDA Special Interest Databases (SIDs) phytonutrient data with USDA SR Legacy. Instructions are specified for the most recent version of data available as of 11/04/2022. NOTE: Future releases may require additional information.

**Files Required**

Files must be collected from five databases: SR Legacy, USDA Database for the Flavonoid Content of Selected Foods, USDA's Expanded Flavonoid Database for the Assessment of Dietary Intakes, USDA Database for the Proanthocyanidin Content of Selected Foods, and USDA Database for the Isoflavone Content of Selected Foods (1,2,3,4,5).

Instructions are provided specifically for the SR Legacy October 2021 JSON release and SR Legacy April 2019 CSV release available on FoodData Central (FDC).

The SIDs should be acquired from the Agricultural Research Service (ARS) website which provides the most recent versions. All four databases are only exportable as MS Access databases; therefore, MS Access 2007 or later is required to open these files.

The most recent version of each SID at the time of writing is as follows: *USDA Database for the Flavonoid Content of Selected Foods, Release 3.3 (March 2018)*, *USDA's Expanded Flavonoid Database for the Assessment of Dietary Intakes, Release 1.1 (December 2015)*, *USDA Database for the Proanthocyanidin Content of Selected Foods, Release 2.1 (March 2018)*, and *USDA Database for the Isoflavone Content of Selected Foods, Release 2.1 (November 2015)*.

From each database, two tables contain variables necessary for integration: “NUTR_DEF” and “FLAV_DAT” from each flavonoid database, “NUTR_DEF” and “PA_DAT” from the proanthocyanidin database and “NUTR_DEF” and “ISFL_DAT” from the isoflavone database. The “NUTR_DEF” files identify names and IDs for each phytonutrient, the tables ending in “_DAT” contain IDs needed to identify foods and the phytonutrient content of those foods. Additional tables in these MS Access files can be used to determine the source and derivation of each phytonutrient measure if needed.

***Joining Information***

There are many different softwares and methods used to join tables, however R is reliable and widely utilized for statistical analysis. For this analysis, tables from each database were imported into R as data frames and joined using the tidyverse library of packages (6, 7). The variable *nutrient number* was used to join the “NUTR_DEF” tables to the “DAT” tables through a series of outer joins, then the variable *nutrient database number* was utilized to combine all databases through a set of full outer joins.

To join tables, differences in variable declarations must be accounted for. In this set of instructions, variables were renamed before implementing joins. The differences in variable declarations found in the necessary files are documented below.

*“NUTR_DEF” Variables:*

- Nutrient Number
  - “Nutr_no” in DB for Flavonoid Content
  - “Nutr_No” in Expanded Flavonoid DB, DB for Isoflavone Content and DB for Proanthocyanidin Content
  - “nutrient.number” or “nutrient_number” in SR Legacy JSON file
  - “nutrient_nbr” in “nutrient” file in SR Legacy CSV from FDC
- Nutrient Name/ Description
  - “Nutrient name” in DB for Flavonoid Content and Expanded Flavonoid DB
  - “NutrDesc” in DB for Isoflavone Content and DB for Proanthocyanidin Content
  - “nutrient.name” or “nutrient_name” in SR Legacy JSON
  - “name” in “nutrient” file in SR Legacy CSV from FDC
- Unit
  - “Unit” in Expanded Flavonoid DB, DB for Flavonoid Content and DB for Isoflavone Content
  - “Units” in DB for Proanthocyanidin Content
  - “nutrient.unitName” or “nutrient_unitName” in SR Legacy JSON
  - “unit_name” in “nutrient” file in SR Legacy CSV from FDC

*“DAT” Variables:*

- Nutrient Database Number
  - “NDB_No” in Expanded Flavonoid DB, DB for Flavonoid Content and DB for Isoflavone Content
  - “NDB No” in DB for Proanthocyanidin Content
  - “ndbNumber” in SR Legacy JSON
  - “NDB_number” in “sr_legacy_food” file in SR Legacy CSV from FDC
- Nutrient Number
  - “Nutr_no” in DB for Flavonoid Content and DB for Isoflavone Content
  - “Nutr_No” in Expanded Flavonoid DB and DB for Proanthocyanidin Content
  - “nutrient.number” or “nutrient_number” in SR Legacy JSON file
  - “nutrient_nbr” in “nutrient” file in SR Legacy CSV from FDC
- Value
  - “Flav_Val” in Expanded Flavonoid DB, DB for Flavonoid Content and DB for Proanthocyanidin Content
  - “Isfl_Val” in DB for Isoflavone Content
  - “Isfl_Val” in DB for Isoflavone Content
  - “amount” in SR Legacy JSON
  - “amount” in “food_nutrient” file in SR Legacy CSV from FDC

**Example Code:**

```{r}

library(jsonlite)

library(tidyverse)

library(readxl)

# Read in data from expanded flavonoid DB

EXP_FLAV_DAT <- read_excel("./EXP_FLAV_DAT.xlsx") %>%

select(NDB_No, Nutr_No, Flav_Val) %>%

rename("Val" = "Flav_Val")

EXP_NUTR_DEF <- read_excel("./EXP_NUTR_DEF.xlsx") %>%

select(Nutr_No, `Nutrient name`, Unit) %>%

rename("nutrient_name" = "Nutrient name")

# Read in data from flavonoid DB

FLAV_DAT <- read_excel("./FLAV_DAT.xlsx") %>%

select(NDB_No, Nutr_no, Flav_Val) %>%

rename("Val" = "Flav_Val") %>%

rename("Nutr_No" = "Nutr_no")

FLAV_NUTR_DEF <- read_excel("./FLAV_NUTR_DEF.xlsx") %>%

select(Nutr_no, `Nutrient name`, Unit) %>%

rename("Nutr_No" = "Nutr_no") %>%

rename("nutrient_name" = "Nutrient name")

# Read in data from isoflavone DB

ISFL_DAT <- read_excel("./ISFL_DAT.xlsx") %>%

select(NDB_No, Nutr_No, Isfl_Val) %>%

rename("Val" = "Isfl_Val")

ISFL_NUTR_DEF <- read_excel("./ISFL_NUTR_DEF.xlsx") %>%

select(Nutr_no, NutrDesc, Unit) %>%

rename("Nutr_No" = "Nutr_no") %>%

rename("nutrient_name" = "NutrDesc")

# Read in data from proanthocyanidin DB

PA_DAT <- read_excel("./PA_DAT.xlsx") %>%

select(`NDB No`, Nutr_No, Flav_Val) %>%

rename("Val" = "Flav_Val") %>%

rename("NDB_No" = "NDB No")

PA_NUTR_DEF <- read_excel("./PA_NUTR_DEF.xlsx") %>%

select(Nutr_No, NutrDesc, Units) %>%

rename("nutrient_name" = "NutrDesc") %>%

rename("Unit" = "Units")

# Read in SR Legacy

SR_json <- jsonlite::fromJSON("./FoodData_Central_sr_legacy_food_json_2021-10-28.json") %>% as.data.frame()

SR_json <- SR_json %>% rename_with(~str_remove(., 'SRLegacyFoods.'))

SR_json <- SR_json %>%

map_if(is.data.frame, list) %>%

as_tibble() %>%

unnest(foodNutrients, keep_empty = TRUE)

SR_json <- unpack(SR_json, cols=c(nutrient), names_sep = "_")

# implement joins

df_def <- EXP_NUTR_DEF %>%

full_join(FLAV_NUTR_DEF) %>%

full_join(ISFL_NUTR_DEF) %>%

full_join(PA_NUTR_DEF)

df_dat <- EXP_FLAV_DAT %>%

full_join(FLAV_DAT) %>%

full_join(ISFL_DAT) %>%

full_join(PA_DAT)

df <- full_join(df_def, df_dat) %>%

distinct() %>%

filter(NDB_No %in% SR_json$ndbNumber)

```

**References**

1. Haytowitz D, Ahuja J, Wu X, Somanchi M, Nickle M, Nguyen Q et al. USDA National Nutrient Database for Standard Reference, Legacy Release [Internet]. Nutrient Data Laboratory, Beltsville Human Nutrition Research Center, ARS, USDA; 2019 [cited 2022 Oct 4]. Available from: https://fdc.nal.usda.gov/
2. Bhagwat S, Haytowitz D B. USDA Database for the Flavonoid Content of Selected Foods, Release 3.3 [Internet]. Nutrient Data Laboratory, Beltsville Human Nutrition Research Center, ARS, USDA; 2018 Mar [modified 2022 Jun 14;cited 2022 Oct 4]. Available from: <https://data.nal.usda.gov/dataset/usda-database-flavonoid-content-selected-foods-release-33-march-2018>
3. Bhagwat S, Haytowitz D B. USDA Database for the Isoflavone Content of Selected Foods, Release 2.1 [Internet]. Nutrient Data Laboratory, Beltsville Human Nutrition Research Center, ARS, USDA; 2015 Nov [modified 2022 May 24;cited 2022 Oct 4]. Available from: <https://doi.org/10.15482/USDA.ADC/1324538>
4. Bhagwat S, Haytowitz D B. USDA Database for the Proanthocyanidin Content of Selected Foods, Release 2 [Internet]. Nutrient Data Laboratory, Beltsville Human Nutrition Research Center, ARS, USDA; 2015 [modified 2022 May 24;cited 2022 Oct 4]. Available from: <https://doi.org/10.15482/USDA.ADC/1324621>
5. Bhagwat S, Haytowitz D B, Wasswa-Kintu S. USDA's Expanded Flavonoid Database for the Assessment of Dietary Intakes, Release 1.1 [Internet]. Nutrient Data Laboratory, Beltsville Human Nutrition Research Center, ARS, USDA; 2015 Dec [modified 2022 May 24;cited 2022 Oct 4]. Available from: <https://doi.org/10.15482/USDA.ADC/1324677>
6. R Core Team. R: A language and environment for statistical computing. Version 4.2.0 [software]. R Foundation for Statistical Computing, Vienna, Austria. 2021 [cited 2022 Nov 4]. Available from: [https://www.R-project.org/](https://www.r-project.org/)
7. Wickham H, Averick M, Bryan J, Chang W, McGowan LD, François R et al. (2019). Welcome to the tidyverse. *Journal of Open Source Software*. 2019; **4**(43), 1686. [doi:10.21105/joss.01686](https://doi.org/10.21105/joss.01686).
